# Point-of-care ultrasound in early diagnosis and monitoring of deep abscess in newborns: a case report of two cases

**DOI:** 10.3389/fped.2024.1325395

**Published:** 2024-05-01

**Authors:** Bang Du, Fengdan Xu, Biying Deng, Baimao Zhong, Ning Li, Xiaoguang He

**Affiliations:** ^1^ Department of Neonatology, Dongguan Children’s Hospital Affiliated to Guangdong Medical University, Dongguan, China; ^2^ Key Laboratory of Newborn Critical Illness, Dongguan Children's Hospital Affiliated to Guangdong Medical University, Dongguan, China

**Keywords:** point-of-care ultrasound, newborn, deep abscess, fever, early diagnosis, monitoring

## Abstract

**Objective:**

This study sought to analyze the value of point of care ultrasound (POCUS) in early diagnosis and monitoring of deep abscess in newborns.

**Methods:**

Retrospective analysis of the clinical data of two newborns admitted to the Neonatal Intensive Care Unit (NICU) of our hospital and diagnosed with deep abscess of the newborn. Combined with literature analysis, the value of POCUS in early diagnosis and monitoring of deep abscess of the newborn was evaluated.

**Results:**

The two newborns reported in this article were all admitted to NICU due to” “fever”. POCUS was used to assist in early diagnosis of “liver abscess” and “lung abscess”. Subsequently, POCUS was used to monitor lesion changes and adjust treatment plans. All patients were cured and discharged with a good prognosis.

**Conclusions:**

Deep abscesses in newborns are very rare and often life-threatening, but apart from fever, they often have no specific clinical manifestations and are easily misdiagnosed or missed. POCUS, as a bedside auxiliary examination tool, has high accuracy, radiation free, non-invasive, and convenient, and has high diagnostic and monitoring value in early diagnosis and monitoring of deep abscess in newborns.

## Introduction

Fever is one of the most common symptoms in newborns seeking medical attention. Some patients have no obvious directional symptoms or signs other than fever, and after preliminary clinical evaluation, they were diagnosed as fever without a source of infection (FWS), also known as fever without localized signs (FWLS) or fever without focal signs (1). Among them, liver abscesses (2–6), lung abscesses (7), kidney abscesses (8, 9), pelvic abscesses (10) and other deep abscesses in newborns are also causes of FWS. In addition to fever, there are often no specific clinical manifestations, which are prone to misdiagnosis and missed diagnosis. If not diagnosed and treated in a timely manner, it may develop into sepsis, and even lead to permanent disability or death, with serious consequences. At present, the diagnostic methods recommended in FWS related guidelines are mainly laboratory tests, such as blood routine, C-reactive protein, procalcitonin, cerebrospinal fluid analysis, blood culture, etc (11, 12). For the diagnosis of deep abscess, except for laboratory tests, imaging examinations such as x-ray, CT, and ultrasound also play an important role. However, considering the issue of radiation exposure, it is not recommended to use x-ray and CT as routine examinations for FWS infants (13). Point of care ultrasound (POCUS) refers to the ultrasound examination performed by a clinical doctor on a patient at the bedside (14). POCUS can conduct real-time longitudinal evaluation as needed, and has the advantages of non-invasive and radiation free, making it an effective tool for identifying potential infectious foci, especially deep abscesses, in newborns with FWS. This study retrospectively analyzed the clinical data of two newborns admitted to the Neonatal Intensive Care Unit (NICU) of our hospital due to FWS and finally diagnosed with deep abscess of the newborn. Combined with literature analysis, the application value of POCUS in early diagnosis and monitoring of deep abscess of the newborn was analyzed.

## Case presentation

### Case 1

The first patient, a male neonate, 26 days old, was admitted to the hospital due to “fever for 2 days”. This patient experienced fever 2 days ago without any obvious cause, with a maximum body temperature of 38.4°C. There was no cough, shortness of breath, restlessness, convulsions, vomiting, diarrhea, or other abnormal symptoms. He had good appetite and normal bowel movements. The baby was born in G2P2, with a gestational age of 38 + 1 weeks. He was born through vaginal delivery and had no history of asphyxia or rescue. The Apgar scores at 1, 5, and 10 min were all 10 points, and the birth weight was 2,660 g. There is no history of infection or other diseases during the perinatal period. Admission examination: T 38.1°C, P 150 times/min, R 50 times/min, BP 81/46 mmHg, no skin bleeding points or rashes, no abnormal signs in the heart, lungs, and abdomen examination, normal muscle tone, normal sucking, foraging, gripping, hugging reflexes, no abnormal neurological signs such as neck resistance.

#### Laboratory examination

White blood cell count (WBC) 23.19 × 10^9^/L (reference value range: 12.0 × 10^9^/L–20.0 × 10^9^/L), Platelet (PLT) 394 × 10^9^/L (reference value range: 125 × 10^9^/L–350 × 10^9^/L), Neutrophils (NEUT) 11.74 × 10^9^/L (reference value range: 1.8 × 10^9^/L–6.3 × 10^9^/L), C-reactive protein (CRP) 132.1 mg/L (reference value range: ≤6.0 mg/L), procalcitonin (PCT) 0.59 ng/ml (reference value range: 0.0–0.5 ng/ml), interleukin-6 (IL-6) 72.9 pg/ml (reference value range: 0.0–7.0 pg/ml), IgA 0.600 g/L (reference value range: 0.003–0.013 pg/ml), IgG 10.500 g/L (reference value range: 5.7–13.7 pg/ml), IgM 0.510 g/L (reference value range: 0.06–0.2 pg/ml), C3 1.020 g/L (reference value range: 0.79–1.52 pg/ml), C4 0.200 g/L (reference value range: 0.16–0.38 pg/ml), no abnormalities were found in cerebrospinal fluid routine and biochemistry, no abnormalities were found in urine routine and fecal routine, and both blood culture results were negative.

#### Auxiliary examination

**Figure 1:**
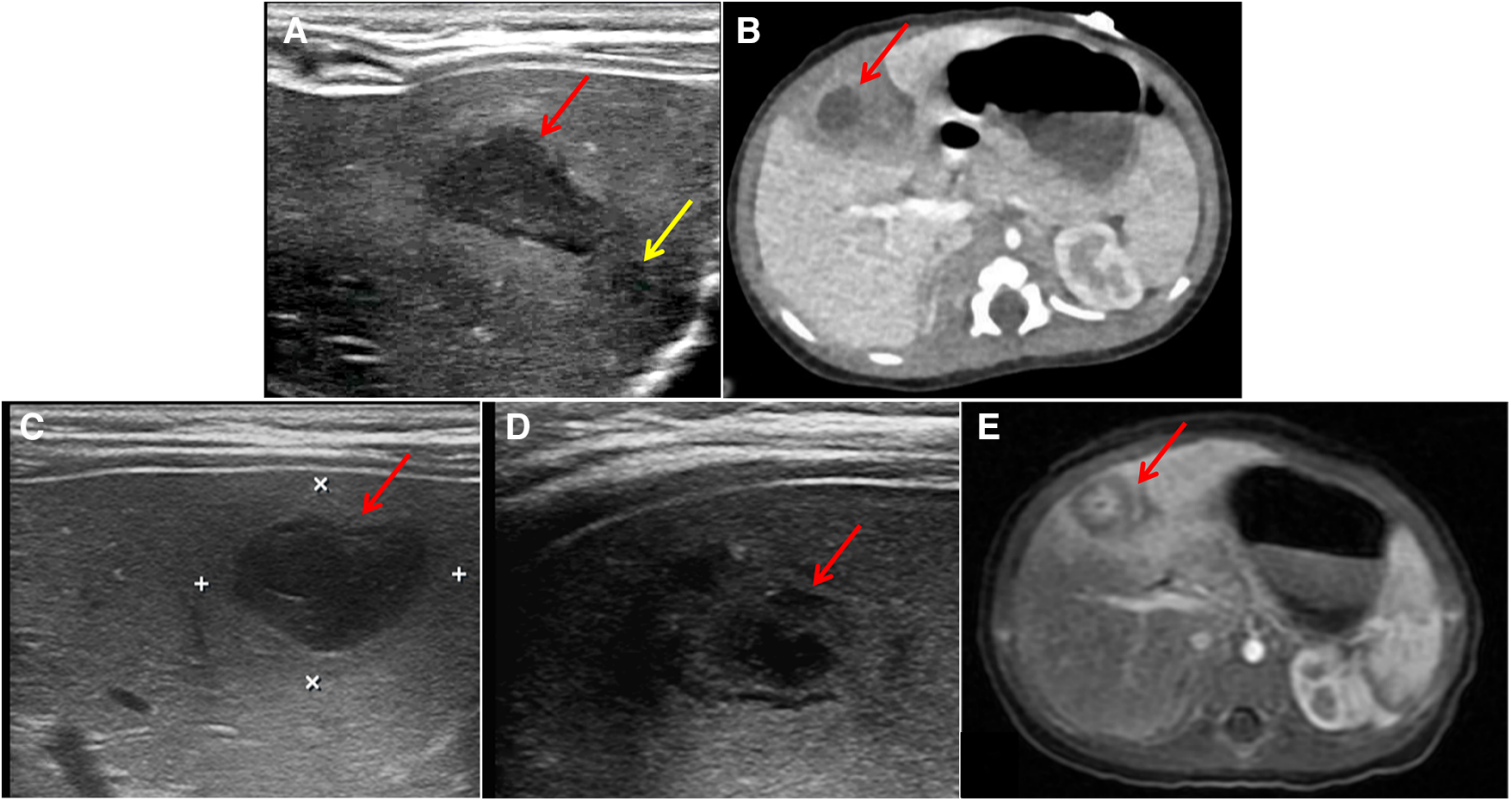
Imaging examination upon admission and reexamination of imaging examination. (**A**) Abdominal ultrasound: Irregular hypoechoic area in the S4 segment of the left lobe of the liver (Red arrow, 21 mm × 10 mm); A low echo mass (Yellow arrow, 5 mm × 5 mm) can be seen next to it, there was no obvious blood flow signal. (**B**) Abdominal CT plain scan and enhancement: liver S4 segment abscess (Red arrow, 29 mm × 36 mm × 33 mm), circular low density non enhanced liquid density shadow with a size of approximately 12 mm × 8 mm can be seen inside it. (**C,D**) Recheck liver ultrasound on the 3rd day and 4th day of admission: no significant change in abscess size (Red arrow, 19 mm × 14 mm, 6 mm × 5 mm) and no new abscess. (**E**) Abdominal MRI plain scan and enhancement on the 21st day of admission: significant absorption of liver S4 segment abscess lesions, with local small pus cavities remaining (Red arrow, 15 mm × 11 mm × 19 mm).

(1) Abdominal ultrasound on the first day of admission prompts: Irregular hypoechoic area can be seen in the S4 segment of the left lobe of the liver, with a range of approximately 21 mm × 10 mm, with clear boundaries and uneven internal echoes. A low echo mass with size of approximately 5 mm × 5 mm can be seen next to it. They seems connected with each orther (Figure 1A). On the second day of admission, abdominal CT plain scan and enhancement: considering liver S4 segment abscess: range 29 mm × 36 mm × 33 mm, circular low density non enhanced liquid density shadow with a size of approximately 12 mm × 8 mm can be seen inside it (Figure 1B).
(2) Recheck liver ultrasound on the 3rd day (Figure 1C) and 4th day (Figure 1D) of admission, respectively. The results showed that no significant change in abscess size (19 mm × 14 mm, 6 mm × 5 mm) and without any new abscess. On the 21st day of admission, abdominal MRI plain scan and enhancement (Figure 1E) showed significant absorption of liver S4 segment abscess lesions (15 mm × 11 mm × 19 mm), with local small pus cavities remaining.

#### Diagnosis and treatments

On the first day of admission, the patient was diagnosed with neonatal liver abscess through POCUS. Empirically, the patient was treated with ampicillin combined with ceftazidime for anti infection. The body temperature returned to normal on the fourth day, and on the 18th day of admission, the infection indicators such as WBC and CRP were normal. The condition improved, and the patient was hospitalized for 25 days and discharged.

#### Follow up and outcome

**Figure 2:**
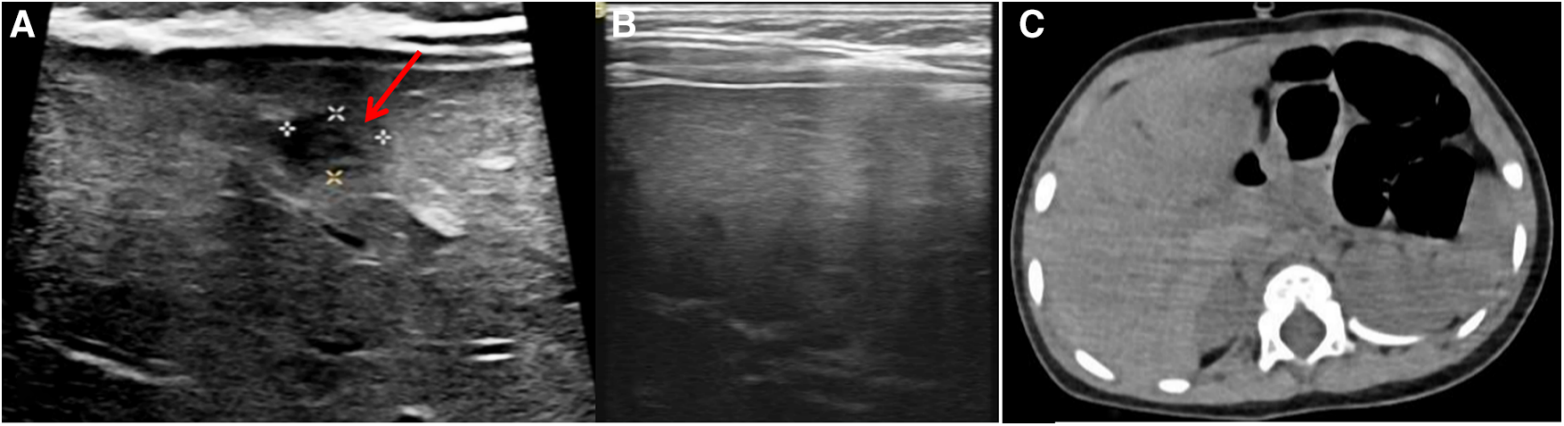
Post-discharge follow-up imaging results. (**A**) Abdominal ultrasound (one month after discharge): liver abscess had shrunk (Red arrow, 3.5 mm × 2.8 mm). (**B**) Abdominal ultrasound (three month after discharge): liver abscess had absorbed. (**C**) Abdominal CT scan (eight month after discharge): no liver parenchymal lesions.

The patient did not experience any further symptoms such as fever after discharge. One month after discharge, the abdominal ultrasound examination showed that the liver abscess had shrunk (3.5 mm × 2.8 mm) compared to before (Figure 2A). After 3 months of discharge, the abdominal ultrasound examination showed that the liver abscess had absorbed (Figure 2B), and the growth and development of the patient were good. After 8 months of discharge, abdominal CT scan showed no liver parenchymal lesions (Figure 2C).

### Case 2

The male patient, 10 days old, was admitted to the hospital due to “fever for 2 days”. The baby began to experience fever within 2 days, with a peak temperature of 38.8°C and accompanied by shortness of breath. The transcutaneous blood oxygen saturation fluctuated between 92% and 96%, and there was no cough, phlegm and low response. He had normal appetite, and normal bowel movements. The baby was born in G3P1, with a gestational age of 38 weeks. He was born through vaginal delivery and had no history of asphyxia or rescue. The Apgar scores at 1, 5, and 10 min were all 10 points, and the birth weight was 3,735 g. There is no history of infection or other diseases during the perinatal period. Admission examination: T37°C, P 153 times/min, R 45 times/min, BP 89/45 mmHg. Clear mind and responsive. No skin bleeding points or rashes were found, the anterior fontanel was flat, the respiratory sounds in both lungs were thick and symmetrical, and no dry or wet rales were heard. The heart rate is 135 beats per min, the heart sound is strong, and the rhythm is synchronized. The abdomen is flat and soft, without touching the mass, without reaching the liver and spleen under the ribs, with bowel sounds of 3 times per min. Sucking, foraging, gripping, and embracing reflexes are normal. No abnormal signs of the nervous system such as neck resistance.

#### Laboratory examination

WBC 22.18 × 10^9^/L (reference value range: 12.0 × 10^9^/L–20.0 × 10^9^/L), PLT 263 × 10^9^/L (reference value range: 125 × 10^9^/L–350 × 10^9^/L), NEUT 14.27 × 10^9^/L (reference value range: 1.8 × 10^9^/L–6.3 × 10^9^/L), CRP 41.66 mg/L (reference value range: ≤6.0 mg/L), PCT 0.17 ng/ml (reference value range: 0.0–0.5 ng/ml), Chlamydia trachomatis DNA positive, 1.32 × 10^3^ copies/ml (reference range: <5 × 102 copies/ml), endotoxin 5.12 pg/ml (reference range: <10.0 pg/ml), fungi (1–3)-β-D-glucan 41.66 pg/ml (reference range: <60.0 pg/ml), negative T cell test for tuberculosis infection (T-SPOT), IL-2 0.01 pg/ml (reference range: 0.00–5.30 pg/ml), IL-4 0.01 pg/ml (reference range: 0.0–2.8 pg/ml), IL-6 1.31 pg/ml (reference range: 0.0–0–7.0 pg/ml), IL-10 1.35 pg/ml (reference range: 0.00–4.91 pg/ml), IFN-γ 1.05 pg/ml (reference range: 0.00–7.42 pg/ml), tumor necrosis factor-α (TNF-α) 0.8 pg/ml (reference range: 0.0–4.6 pg/ml), both blood cultures were negative, urine culture negative.

#### Auxiliary examination

**Figure 3:**
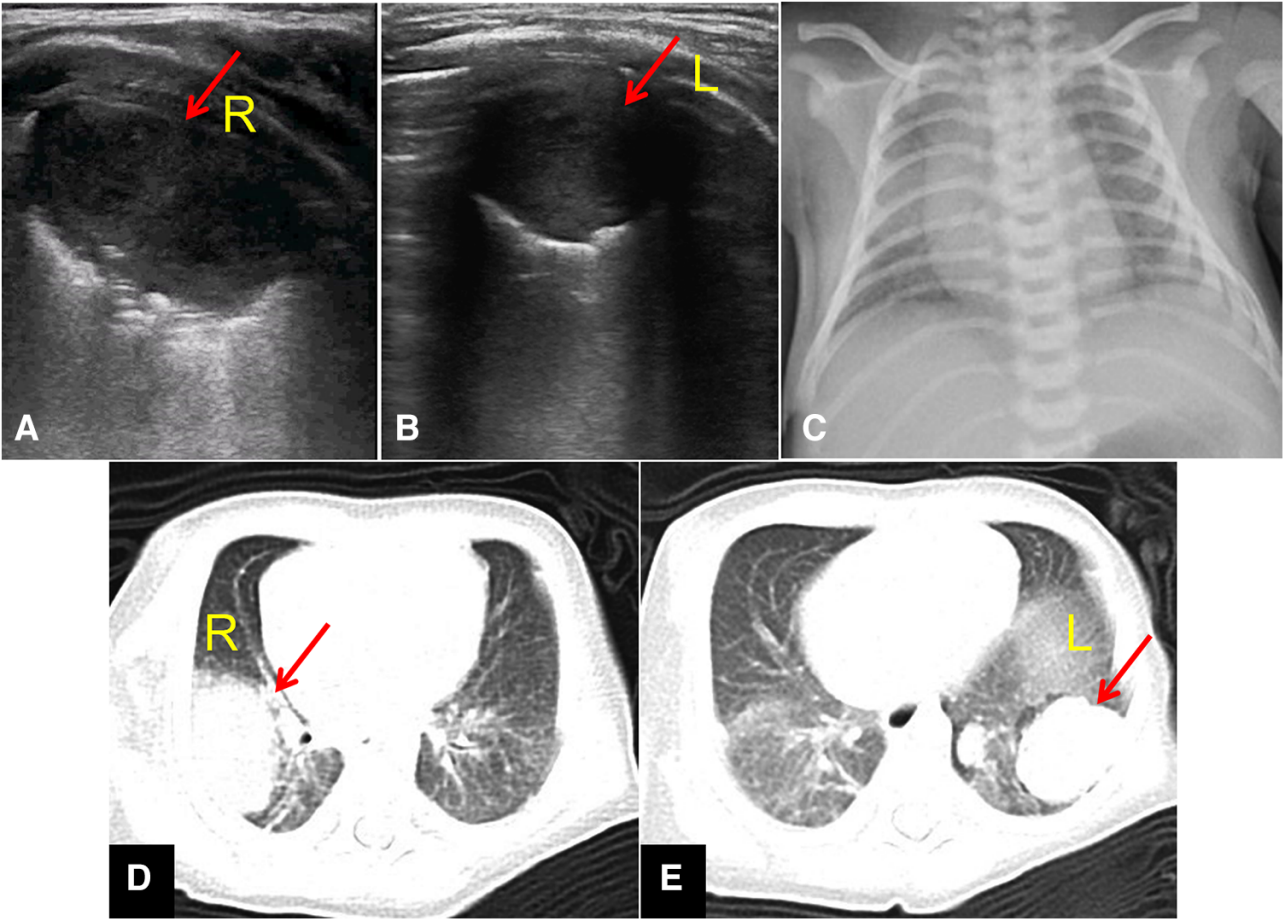
Imaging examination upon admission. (**A**) Lung ultrasound: elliptical consolidation lesions in the right axillary area (Red arrow, 32.3 mm^3^ × 20.2 mm^3^ × 18.1 mm^3^). (**B**) Lung ultrasound: elliptical consolidation lesions in the left lower lung (Red arrow, 22.2 mm^3^ × 16.4 mm^3^ × 15.8 mm^3^). (**C**) Chest x-ray examination: indicating right pneumonia. (**D**) An elliptical soft tissue mass shadow in the posterior segment of the right upper lobe (Red arrow, 31 mm^3^ × 22 mm^3^ × 20 mm^3^), with a large necrotic area in the center. (**E**) Multiple nodules and lumpy shadows in the lower lobe of the left lung (Red arrow, up to approximately 25 mm^3^ × 22 mm^3^ × 15 mm^3^).

On the second day of admission, lung ultrasound examination revealed elliptical consolidation lesions in the right axillary area and the dorsal side of the left lower lung (the size of the lesion in the right lung is about 32.3 mm^3^ × 20.2 mm^3^ × 18.1 mm^3^, left lung lesion size approximately 22.2 mm^3^ × 16.4 mm^3^ × 15.8 mm^3^), considering the possibility of lung abscess (Figures 3A,B). Upon admission, a chest x-ray examination was performed simultaneously, indicating right pneumonia (Figure 3C).

On the 3rd day of admission, chest CT showed multiple lung abscesses in both lungs: an elliptical soft tissue mass shadow in the posterior segment of the right upper lobe, approximately 31 mm^3^ × 22 mm^3^ × 20 mm^3^, with a large necrotic area in the center; Multiple nodules and lumpy shadows in the lower lobe of the left lung, up to approximately 25 mm^3^ × 22 mm^3^ × 15 mm^3^, with enhancement resembling a right lung mass (Figures 3D,E).

#### Diagnosis and treatments

**Figure 4:**
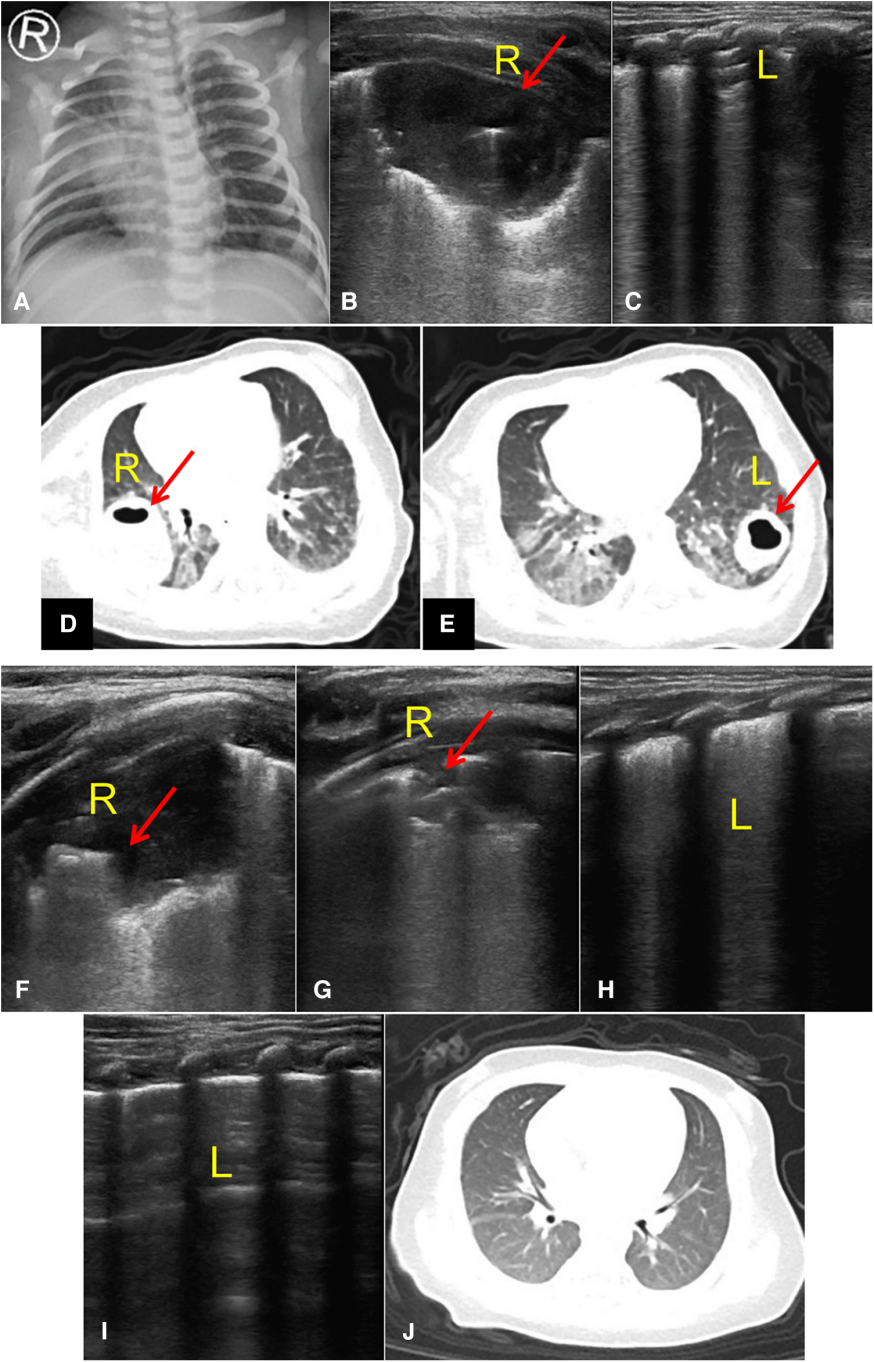
Post-treatment follow-up imaging results. (**A**) Chest x-ray showed an improvement in pneumonia compared to before. (**B,C**) Lung ultrasound: an irregular small consolidation (Red arrow, 25.8 mm^3^ × 12.2 mm^3^ × 10.3 mm^3^) in the posterior axillary area of the right upper lung, a small amount of bronchial inflation sign is visible, and no significant consolidation is detected in the rest of the right lung and left lung. (**D,E**) Chest CT scan: lumpy shadows in both lungs, with a right lung lesion size of approximately 15 mm^3^ × 20 mm^3^ × 28 mm^3^, with a left lung lesion size of approximately 20 mm^3^ × 17 mm^3^ × 10 mm^3^, smaller than before. (**F–I**) Lung ultrasound: an irregular small consolidation (Red arrow, 12.2 mm × 8.6 mm) in the posterior axillary area of the right upper lung, with a small amount of bronchial inflation sign visible. No significant consolidation was detected in the remaining areas of the right lung and left lung (**J**) Chest CT scan: patchy slightly high-density shadows in the upper and lower lobes of the right and left lungs, the lesions in both lungs are significantly absorbed and reduced compared to before, and the cavities disappear.

Upon admission, pulmonary abscess was diagnosed and treated with intravenous infusion of ampicillin (50 mg/kg Q8h for 3 days), oral administration of azithromycin for anti infection (10 mg/kg qd for 5 days), intravenous infusion of methylprednisolone (0.26 mg/kg Qd for 5 days), and infusion of fresh frozen plasma (13 ml/kg for 2 days). After one week of treatment, the chest x-ray showed an improvement in pneumonia compared to before (Figure 4A). Lung ultrasound (Figures 4B,C) shows an irregular small consolidation with a size of approximately 25.8 mm^3^ × 12.2 mm^3^ × 10.3 mm^3^ in the posterior axillary area of the right upper lung, a small amount of bronchial inflation sign is visible, and no significant consolidation is detected in the rest of the right lung and left lung. Chest CT scan shows lumpy shadows in both lungs, with a right lung lesion size of approximately 15 mm^3^ × 20 mm^3^ × 28 mm^3^, smaller than before (Figures 4D,E). The patient was hospitalized for 10 days and discharged with stable condition.

#### Follow up and outcome

One month after discharge, lung ultrasound examination revealed that an irregular small consolidation with a size of approximately 12.2 mm × 8.6 mm can be seen in the posterior axillary area of the right upper lung, with a small amount of bronchial inflation sign visible. No significant consolidation was detected in the remaining areas of the right lung and left lung (Figures 4F–I). Chest CT scan shows patchy slightly high-density shadows in the upper and lower lobes of the right and left lungs. The lesions in both lungs are significantly absorbed and reduced compared to before, and the cavities disappear (Figure 4J).

## Discussion

Neonatal deep abscesses are a critical illness that requires timely screening, diagnosis, and treatment. However, in clinical practice, except for fever, deep abscesses often lack specific symptoms and signs, making them prone to misdiagnosis and missed diagnosis. The two cases of deep abscess in newborns analyzed in this article were liver abscess and lung abscess, both of which were diagnosed early with POCUS during hospitalization. During the treatment process, POCUS was used for dynamic monitoring to guide the adjustment of treatment plans and achieve good prognosis.

Intra-abdominal abscess is a relatively rare clinical condition. It is reported that the incidence of intra-abdominal abscess is about 0.031% in children admitted to emergency departments, with liver abscess being a rare lesion (15). The microorganisms that cause abscesses may reach the liver through ascending infection, hematogenous transmission, and direct dissemination from adjacent organs through the umbilical vein, portal vein, or biliary tract. The patient had no history of the above diseases, and blood culture, urine culture, stool culture and cerebrospinal fluid culture during hospitalization were negative, and no etiology and no evidence of infection of organs other than the liver were found. And immunoassay was no obvious abnormalities, which does not support immune deficiency diseases. Its most common manifestation is an explosive course of disease, characterized by systemic sepsis and multiple metastatic abscesses, with a hidden condition (16). Symptoms can be non-specific, such as fever, feeding intolerance, vomiting, bloating, liver enlargement, etc. Laboratory test results such as increased white blood cells, neutropenia, thrombocytopenia, increased erythrocyte sedimentation rate, and elevated liver enzymes are also non-specific (2, 17). The diagnosis of intra-abdominal abscess is largely based on clinical knowledge of newborns with sepsis of unknown causes and lesions, especially those with a history of abdominal or vascular catheterization (18). Ultrasound is an effective, non-invasive, economical, and radiation free auxiliary examination method (19), and has become an attractive tool for evaluating critically ill patients. It can be easily operated by intensive care unit doctors at the bedside (20). Abdominal ultrasound is the preferred examination for liver abscess, and its sensitivity is reported to be between 80% and 90% in adult patients (21). Case 1 of this article was diagnosed as a liver abscess with fever as the main manifestation. However, the symptoms and signs related to liver infection were not specific in the early stage, and the condition was hidden. Ultrasound screening was used to screen the lesion, indicating abnormal echoes in the left lobe of the liver, manifested as irregular hypoechoic areas with clear boundaries and uneven internal echoes. Further improve abdominal CT to clarify liver S4 segment abscess. During the subsequent treatment process of the patient, POCUS was also used to dynamically monitor the lesion and guide the adjustment of treatment plan. There is still controversy over whether abscess puncture is necessary for neonatal liver abscess. In this case, considering that the boundary of the neonatal abscess is not reinforced, the capsule is not obvious, and puncture does not exclude the risk of bleeding, worsening of infection, and the patient's body temperature is stable after intravenous anti infection treatment. On the 3rd, 7th, and 14th days of anti infection treatment, the liver abscess lesion is monitored as before, with no increasing trend, and there are no complications such as pneumonia, pleurisy, subphrenic or liver abscess. Based on relevant literature reports suggesting the rehabilitation of premature infants with liver abscess after intravenous anti infection (2), conservative treatment was ultimately decided. Monitoring revealed that the abscess lesion in the patient gradually shrank and no new abscess lesions were found. Since no puncture or drainage was performed and no pus culture results were obtained, it was finally considered that the source and etiology of the liver abscess in this case was unknown. After 3 months, the patient's liver abscess was completely absorbed and the prognosis was good. The patient was successfully treated conservatively, which can be explained by early diagnosis and effective anti-infective therapy. It is also suggested that the occurrence of neonatal liver abscess can be without any clearly identified predisposition factors. For children with uncertain infection site or who do not respond to conventional treatment, the possible presence of liver abscess should be evaluated by abdominal liver ultrasound and antibiotic treatment should be designed as soon as possible.

Lung abscess is also a relatively rare pediatric disease, with a incidence rate as low as 0.7/100.000 hospital admission/year (22). The incidence rate of lung abscess in children is also lower than that in adults (23). It is even rarer in newborns (24). There are research reports that fever, cough, and difficulty breathing are the most common symptoms of lung abscess (25, 26), and the symptoms are not typical in newborns, with fever being the main symptom. The main symptom of case 2 in this article is fever, accompanied by shortness of breath, and the initial diagnosis is infectious pneumonia. On the first day of admission, POCUS examination was performed and abnormal lesions were found in the lungs, characterized by consolidation in both lungs. One consolidation lesion was detected in both lungs, with an elliptical shape, non serrated, and liver like echo. However, the echo was uneven, and no bronchial inflation sign was observed. The boundary between the consolidation area and other lung tissues was clear, and the diagnosis was lung abscess. Subsequent CT examination confirmed multiple lung abscesses in both lungs, and the location and size of the abscess were similar to the ultrasound monitoring results. The patient underwent synchronous chest x-ray examination upon admission, and the results reported that the patient had right pneumonia. However, the x-ray examination did not show any changes of lung abscess. The possible reasons may be: the x-ray manifestations of pneumonia and lung abscess are similar, they are often difficult to be distinguished; x-rays cannot be taken from multiple angles, making it difficult to detect hidden lesions. Lung ultrasound has certain advantages in the examination of various lung diseases (27). Clara Kraft et al. (28) reported a case of pediatric pulmonary abscess, which also showed elliptical consolidation on ultrasound, with a surrounding hyperechoic capsule. In addition, some authors suggest that further identification of the presence of pneumothorax is necessary when a lung abscess is discovered. Lung ultrasound can distinguish pneumothorax, pulmonary edema, pulmonary consolidation, atelectasis, etc. based on changes in the ratio of liquid to air in lung lesions (29, 30), the examination results are similar to those of CT. According to Chen's et al. (31) research definition, its sensitivity for diagnosing lung abscess is 94% and specificity is 100%. It is an important tool for diagnosing respiratory diseases and can provide more detailed details than chest x-ray (32). Combined with literature reports (33), The common etiology of pulmonary abscess is *Staphylococcus aureus* and *Streptococcus pneumoniae*, so before the etiology was not clear, ambenicillin was empirically given intravenously for anti-infection. On the third day, the throat swab was positive for chlamydia trachomatis-DNA, and azithromycin was changed to oral anti-infection. If conservative treatment is successful, surgical intervention is not necessary (22). Methylprednisolone and fresh frozen plasma were given along with antibiotic treatment. They act as anti-inflammatory agents (34, 35). In this case, the effect of antibiotic treatment was significant. During hospitalization and after discharge, B-ultrasound and CT of the lungs were closely monitored, and the abscess gradually shrank, so there was no surgical intervention. The literature reports that the time range of intravenous antibiotic use in children with pulmonary abscess is large, the shortest 3 days (33). In this case, intravenous antibiotics were used for 3 days and oral antibiotics for 5 days. During the subsequent treatment process of the patient, POCUS continued to be used to monitor lung abscess lesions, indicating that bilateral lung lesions were still visible, but the number of lesions did not increase and the scope gradually narrowed; The chest CT examination showed that the abscess lesions in both lungs were more absorbed than before. However, a chest x-ray examination did not reveal any left lung abscess lesion. It suggests that using POCUS for monitoring lung abscess lesions may be better than x-ray. According to the literature, most children with pulmonary abscesses recovered smoothly without significant long-term pulmonary sequelae, 51.21% of cases recorded complete radiological regression, and the mean time to radiological regression was 84.14d ± 51.57d (36). This case of pulmonary abscess was similar to the case of liver abscess, except for fever and shortness of breath, no other sites of infection were found. Only the throat swab was positive for chlamydia trachomato-DNA, and the condition was improved after anti-infection treatment, and the abscess was gradually reduced by ultrasonic monitoring. Considering the risk of puncture and the possibility of infection spread, lung abscess puncture and lung bronchoscopy irrigation fluid culture were not performed in the end, and there was no pus culture result. Therefore, the lung abscess in this case was suspected to be caused by lung chlamydia trachomato-infection.

## Conclusion

In summary, the symptoms of deep abscess in newborns are often non-specific and difficult to diagnose. Compared with standard clinical evaluations, POCUS has advantages such as no radiation damage, portability for bedside use, and allows for repeated non-invasive screening in suspected cases. It helps to quickly identify lesions, diagnose early, and also plays an important role in dynamic monitoring of lesions, which is worthy of further clinical promotion and application.

## Data Availability

The original contributions presented in the study are included in the article/Supplementary Material, further inquiries can be directed to the corresponding authors.
